# Clinico-Pathological Characteristics and Mutational Analysis of Gastrointestinal Stromal Tumors from India: A Single Institution Experience

**DOI:** 10.31557/APJCP.2019.20.10.3051

**Published:** 2019

**Authors:** Sachin Minhas, Sunita Bhalla, Mayank Jauhri, Madhusudan Ganvir, Shyam Aggarwal

**Affiliations:** 1 *Department of Medical Oncology,*; 2 *Department of Pathology, Sir Ganga Ram Hospital, New Delhi, India. *

**Keywords:** GIST, Oncology, C-KIT, Gastric, CD117

## Abstract

**Background:**

Gastrointestinal stromal tumor (GIST) is the most common type of mesenchymal neoplasm of gastrointestinal tract. The incidence of GIST in India is not known and its treatment strategy in our country is largely derived from studies in other global populations. Some of the most important features of this type of cancer include its size, site of origin, mitotic index, histology and Immunohistochemistry. In this report we have studied these parameters in the Indian GIST patients presenting at our center. Additionally, we have also studied the mutational spectrum of these GISTs by next generation sequencing.

**Methods:**

Thirty one Indian patients of GIST were enrolled in this study and information regarding age, gender, tumor location and size was collected from their records. Immunohistochemistry studies were performed by the pathologist. Mutational analysis of these samples was performed by next generation sequencing.

**Results and Discussion:**

The most common site of GIST occurrence in our study was stomach. The tumor size for all 31 patients ranged between 0.6 cms to 20 cms. A spindle-cell pattern was present in 24 out of 31 of the cases. 29 out of 31 subjects were positive for *CD117* expression. C-KIT was the most highly mutated gene indentified in our patients. Apart from these findings we observed many similarities as well as dissimilarities between the results of our study and literature published previously.

**Conclusions:**

The dissimilarities in the results of our study and published literature could be attributed to the genetic or ethnic differences that exist between the Indian population and other global populations. The results of our study warrant a need to conduct studies of GIST in a much larger population of India. Such large scale studies may also help in better treatment and/or prevention of GIST in developing countries like India.

## Introduction

Gastrointestinal stromal tumor (GIST) is the most common type of mesenchymal neoplasm of gastrointestinal tract (Steigen and Eide, 2009). It is believed that this cancer arises from the (or precursors of the) interstitial cells of Cajal which are involved in gastrointestinal motility. GIST normally occurs after the age of 40 although a few rare cases in children and young adults have also been reported (Zhao and Yue, 2012). There is no difference between genders with respect to its incidence. Although GISTs may arise anywhere throughout the gastrointestinal tract, the most common site for GIST is stomach (60 %) followed by small intestine (35%) and less than 5% GISTs occur in the rectum, esophagus, omentum, and mesentery (Miettinen and Lasota, 2006). 

The size of the primary tumor and the mitotic index are the most reliable prognostic factors for GISTs. Generally, patients with GISTs greater than or equal to 10 cm in size have a high chance of developing tumor recurrence. On the other hand, those with GISTs less than 2 cm have better prognosis and are more likely to be cured by surgical resection of the tumor. If the number of mitotic cells is less than one per high-power field (HPF) the tumor is considered benign and potentially malignant if there are between one and five mitoses per HPF. It is considered malignant if there are more than five mitoses per HPF (Joensuu et al., 2002; Al Jiffry et al., 2015). In addition to the tumor size and mitotic index the site of tumor also plays a role in prognosis and the reports suggest that stomach GISTs have a better prognosis than small intestine or rectal GISTs (Miettinen and Lasota, 2006; Zhao and Yue, 2012).

Histologically three types of GISTs are seen. The most commonly seen type is the spindle cell type (70%) followed by epithelioid type (20%) and the rest of them (10%) are mixed spindle cell and epithelioid type. Immunohistochemically, the most important marker in GIST is *CD117* (KIT) and approximately 95 % of GISTs are strongly and diffusely positive for the expression of *CD117* protein (Zhao and Yue, 2012). Another marker for GISTs is CD34 but it is not as sensitive or specific as *CD117* (Zhao and Yue, 2012). Thus, *CD34* expression is neither required for GIST diagnosis nor it is unique to GISTs. 10% to 47% of GISTs show expression of smooth muscle actin protein (*SMA*) (Turner and Goldsmith, 2009). The expression of *SMA* is not related to the expression of *CD117*. According to the previous literature the majority of SMA positive GISTs are localized in the small intestine, whereas *SMA*-negative GISTs are more commonly found in the stomach (Yakovenko et al., 2014). 

Like most other cancers the mutational profile of a GIST is an extremely important aspect of it. 70-80% of GISTs harbor mutations in the *KIT* gene and 8-10% of the GISTs have mutations in the *PDGFRA* gene. Mutations in these two genes are known to be mutually exclusive to each other. Within *KIT *gene mutations the mutations of exon 11 are the most commonly occurring mutations followed by the mutations in Exon 9, 13 and 17. Both the KIT and PDGFRA proteins are closely related receptor tyrosine kinases (Rubin et al., 2007; Zhao and Yue, 2012). *PDGFRA* genetic mutations are normally found in gastric GISTs and GISTs with epithelioid morphology (Lasota and Miettinen, 2008). They also follow a lesser malignant course of this disease (Lasota and Miettinen, 2008). Targeted treatment with tyrosine kinase inhibitors (TKI) forms the basis of GIST medical treatment as the conventional chemotherapy and radiotherapy is largely ineffective in this cancer (Rubin et al. 2007). GISTs which do not have either a *KIT* or *PDGFRA* mutation are sometimes referred to as “wild-type” GIST (wt-GIST). They constitute up to 8-10% of total GIST cases and usually contain oncogenic mutations in other genes like *BRAF*, *KRAS*, *NF1* etc. (Zhao and Yue, 2012). 

As per the literature GIST accounts for less than 1% of all gastrointestinal tumors (Zhao and Yue, 2012). The incidence of GIST in India is not known and its treatment strategy in our country is largely derived from studies in other global populations (Shrikhande et al., 2014). Thus there is a strong need to conduct research studies on GIST in India to derive data that is specific to the population of this country. The aim of our study is to present the pathological and histological features, of GIST patients diagnosed at our center. Additionally, we also studied the mutational spectrum of these tumors by next generation sequencing (NGS). 

## Materials and Methods

The ethics committee of our institute approved the study. Written informed consent was obtained from all the subjects included in this study. Thirty one patients of GIST were enrolled. Information regarding age, gender, tumor location and size was collected. 22 subjects were males. 9 subjects were females. The subjects ranged from 29 to 77 years. Formalin-fixed and paraffin-embedded tumor samples were examined. Immunohistochemistry studies were performed by the pathologist for all 31 patients. Mutational analysis was possible in 25 out of 31 patients. Mutational analysis of these samples was performed at two centers (MedGenome Labs Ltd., Bengaluru, India and Strand Life Sciences, Bengaluru, India) and both the centers used the Illumina sequencing platform for NGS analysis (Illumina, San Diego, CA). The tumor DNA of all 25 patients was isolated and subjected to NGS analysis on pre-designed cancer panels with optimized oligonucleotide probes for sequencing mutational hotspots. The Illumina TruSight Tumor 170 next-generation sequencing assay and the Illumina TruSeq Amplicon - Cancer Panel were used for NGS analysis by methods described previously by the manufacturer (Illumina). 

## Results

The most common site of GIST occurrence in our study was stomach (13/31), closely followed by small intestine (11/31). The other sites were colon, retro-peritoneum, abdominal wall and between rectum and vagina. The tumor size for all 31 patients ranged between 0.6 cms to 20 cms. 20 tumors were found to be <10 cm in diameter and 11 tumors were ≥10 cm. The mitotic rate was <5/50 HPF in 18 out of 31 cases and ≥5/50 HPF in rest of the 13 cases. A spindle-cell pattern was present in 24 out of 31 of the cases, epithelioid in 2 out of 31 and a mixed pattern in rest of the 5 cases. 29 out of 31 subjects were positive for *CD117* expression. 2 of 31 subjects were negative for *CD117* expression. 15 out of 31 subjects showed positive expression for SMA and the rest were negative for *SMA *expression.

**Table 1:** Clinico-pathological Characteristics and Results of Mutational Analysis

Subject Number	Sex	Age	Microscopy & Immunohistochemistry				Tumor Characteristics		Mutated Gene	Mutation Description (Amino Acid change)
			CD117	SMA	Mitosis/50 HPF	Histology (cell shape)	Site	Size (cm)		
001	M	29	+	+	3-4	Mixed	Small Intestine	9	KIT	p.Lys642Glu (Exon 13)
002	M	51	+	+	0	Spindle	Small Intestine (Jejunum)	4	Nil	No Mutations Found
003	M	56	+	+	6	Spindle	Small Intestine (Jejunum)	6.5	KIT	p.Lys558_Ile571del (Exon 11)
004	M	37	+	-	5-6	Spindle	Colon	9	KIT	p.Met552_Trp557del (Exon 11)
005	F	58	+	-	2	Spindle	Between Rectum and Vagina	10	KIT	p.Leu576Pro (Exon 11)
									KIT	p.Ala781Val (Exon 16)
006	F	53	+	-	25-30	Spindle	Stomach	12	KIT	p.Trp557_Val559delinsPhe (Exon 11)
007	F	57	+	+	> 40	Spindle	Gall Bladder	7.5	KRAS	p.Gly12Val (Exon 2)
008	M	49	+	+	5	Spindle	Stomach	8	PDGFRA	p.Asp842Val (Exon 18)
009	M	52	+	-	2	Spindle	Stomach	3.5	PDGFRA	p.Asp842del (Exon 18)
									PIK3CA	p.Arg108His (Exon 2)
010	M	62	+	-	2	Spindle	Stomach	14	KIT	p.Ala502_Tyr503dup (Exon 9)
									TP53	p.Arg175His (Exon 5)
011	F	69	+	-	45-48	Epithelioid	Small Intestine (Duodenum)	12	CDKN2A	p.Thr18_Ala19del (Exon 1)
									TP53	p.Cys176Tyr (Exon 5)
									AKT1	p.Ala399Thr (Exon 11)
									ERBB2	p.Arg103Gln (Exon 3)
012	M	45	+	+	3-5	Spindle	Small Intestine (Jejunum)	6.5	KIT	p.Trp557Arg (Exon 11)
									TP53	p.Pro152Leu (Exon 5)
013	M	76	+	-	2-3	Spindle	Small Intestine	0.8	KIT	p.Trp557_Lys558del (Exon 11)
									TP53	p.Cys182Tyr (Exon 5)
									BRAF	p.Arg354Gln (Exon 8)
									PTEN	p.Asp19Asn (Exon 1)
014	M	43	+	+	3-4	Spindle	Colon	16	PDGFRA	p.Thr474Met (Exon 10)
									BRAF	p.Thr401Ile (Exon 10)
015	M	45	+	-	6	Mixed	Stomach	20	PIK3CA	p.Arg916Cys (Exon 19)
016	M	64	+	+	5	Mixed	Small Intestine (Jejunum)	4.5	KIT	p.Val559Ala (Exon 11)
017	M	35	+	-	2-3	Spindle	Stomach	0.4	KIT	p.Trp557_Val559delinsPhe (Exon 11)
018	F	64	+	-	> 40	Spindle	Stomach	19	KIT	p.Met541Leu (exon 10)
									KIT	p.Val559Phe (Exon 11)
									TP53	p.Pro72Arg (Exon 4)
019	M	52	+	-	< 1	Spindle	Retro-Peritoneum	15	NIL	No Mutations Found
020	F	30	+	+	< 1	Epithelioid	Stomach	0.6	KIT	p.Met541Leu (Exon 10)
									KDR	p.Gln472His (Exon 11)
021	M	52	+	+	< 1	Spindle	Stomach	4.5	KIT	p.Trp557Arg (Exon 11)
022	M	44	-	+	< 1	Spindle	Small Intestine	5	CDKN2A	p.Met53Ile (Exon 2)
									TP53	p.Pro72Arg (Exon 4)
023	M	74	+	-	> 40	Mixed	Abdominal Wall	8	KIT	p.Met541Leu (Exon 10)
024	M	53	+	+	3-4	Spindle	Stomach	9	KIT	p.Met541Leu (Exon 10)
									TP53	p.Pro72Arg (Exon 4)
025	M	51	+	-	> 10	Mixed	Small Intestine (Duodenum)	9.5	N.A*	----
026	M	59	-	+	0	Spindle	Small Intestine (Duodenum)	2	N.A*	----
027	F	30	+	-	2	Spindle	Stomach	10	NIL	No Mutations Found
028	M	77	+	+	> 10	Spindle	Stomach	14	N.A*	----
029	F	63	+	-	2-5	Spindle	Stomach	4	N.A*	----
030	F	44	+	+	6	Spindle	Liver	12	N.A*	----
031	M	55	+	-	< 1	Spindle	Small Intestine (Duodenum)	3.5	N.A*	----

* Mutational analysis not possible due to insufficient tumor/bad sample quality.

C-KIT was the most highly mutated gene indentified in our patients (15/25). Mutations in Exon 11 were the most prominent mutations among all the *C-KIT* mutations (10/15). Within the exon 11 mutation carriers group 5 out of 10 patients had exon 11 deletions. The rest of the 5 patients had point mutations in exon 11.* PDGFRA* gene was mutated in 3 out of 25 patients. *PDGFRA* and *C-KIT* mutations were found to be mutually exclusive with respect to each other. 11 out of 25 patients had more than one mutation in their tumor (concomitant mutations). The second most mutated gene after *C-KIT* was *TP53*. The rest of the genes which were found to be mutated included the *KRAS, CDKN2A, AKT1, ERBB2, BRAF*, *PTEN* and *KDR* genes. 7 out of 25 patients had wt-GIST as they did not have any mutation in *KIT* or *PDGFRA *genes. 4 out of these 7 patients had mutations in *KRAS, CDKN2A, TP53, AKT1, ERBB2 *and *PIK3CA* genes. No mutations were detected in rest of the 3 patients. The results of our study are shown in Table 1.

## Discussion

We observed a few dissimilarities between our data and the data already published from other global populations. The incidence of GIST in the stomach was 42% (13/31) in our study (instead of 60% found in previously published data). There was also a single case of a 44 year old female with GIST arising in liver which is notable since gastrointestinal stromal tumors from liver are very rare. The epithelioid type of GIST constituted only 7% of our data instead of 20% as previously reported in the literature. Two subjects were found to be negative for *CD117* expression which is also a notable observation since GIST patients with negative *CD117* expression are very uncommon. KIT mutation was approximately 60% in our patient group (15\25) instead of 70% found in other populations. Similarly wt-GIST was 28% instead of 10% found elsewhere. Mutated *PDGFRA* gene is normally more prevalent in gastric GISTs but we had one patient with *PDGFRA* mutation in colon GIST. We also noticed that all three patients in our study who had *PDGFRA* mutations had spindle type of GIST (instead of epithelioid). According to previous literature* BRAF* mutations generally do not occur with *KIT* or *PDGFRA* mutations (Rossi et al., 2016) but in our group one patient had a *BRAF* mutation along with a *KIT* mutation and another patient harboured a *BRAF* mutation along with a *PDGFRA* mutation. A previous study by Mavroeidis et al., (2018) has also documented the occurrence of such concomitant mutations.

Besides the dissimilarities discussed above we also observed the following similarities between the results of our study and the results published previously. The most common site of occurrence in our patients was stomach which is consistent with other populations. *CD117* expression incidence was similar in our data and previously published data (93% vs 95%). *KIT* mutations were the most common mutations in our group. Also, both *KIT* and *PDGFRA* mutations were found to be mutually exclusive to each other as previously reported in the literature. 

In conclusion, we have described in this study our single centre experience of *GIST* and we have reported a few similarities as well as dissimilarities between the results of our study and literature published previously. The dissimilarities in results could be attributed to the genetic or ethnic differences that exist between the Indian population and other global populations. Although the sample size of our study was small, the results warrant a need to conduct such studies of GIST in a much larger population of our country.
